# Co-development of Happiness Research: Addition to “Fifty Years After the Social Indicator Movement”

**DOI:** 10.1007/s11205-017-1554-z

**Published:** 2017-02-25

**Authors:** Ruut Veenhoven

**Affiliations:** 1 Erasmus Happiness Economics Research Organization, Erasmus University Rotterdam, Rotterdam, The Netherlands; 2 Opentia Research Program, North-West University, Vanderbijlpark, South Africa

**Keywords:** Science history, Happiness, Life satisfaction

## Abstract

Social Indicators Research covers many topics, which each have their own history. Happiness research is one of these included topics. Longstanding interest in happiness revived since the 1960s together with the emergence of the social indicator movement. Happiness became a prominent issue in the movement and the movement has fostered the development of happiness research in several ways.

## Introduction

Social Indicators Research (SIR) can be seen as a *rope*, consisting of several *strands*. This metaphor nicely illustrates: (1) that Social Indicators Research covers many topics, (2) that these topics are intertwined, (3) that they strengthen each other and (4) that together they are stronger than each strand taken separately.

What are these constituting ‘strands’? In their lucid review of the last 50 years of Social Indicators Research Land and Michalos (2017) mention topics such as ‘inequality’, ‘poverty’, ‘safety’ and ‘social cohesion’. ‘Social’ indicators have been used to cover all qualities of life that are not typically ‘economic’, since the movement emerged in the 1960s in reaction to the limitations of mere economic indicators, which dominated research and policy at that time.

‘Happiness’ is one of the strands in Social Indicators Research. Below I will add some detail about this particular research line and sketch the role of happiness research in the wider movement.

## Common Roots

Social Indicators Research roots in the view that human society can be improved using scientific knowledge. This view emerged in the eighteenth century as part of the European ‘Enlightenment’. This intellectual movement contested several views common in the ‘dark’ middle ages.

One of these contested views was that society is a moral order given by God, which humans cannot and should not change. Enlightened thinkers saw society to be a result of human contracts, which can be revised and should be changed if these appear to involve undesirable consequences. Taking a, still prevalent, religious perspective, they emphasized a moral obligation to perfect ‘God’s garden’ on earth and too weed out injustice.

Another challenged view was that earthly life is not to be enjoyed, since man has been expelled from Paradise and that happiness will only be possible in an afterlife in Heaven, if only for the chosen. Enlightened thinkers rejected the view of a punishing God and rather believed in a loving God, who likes to see his children enjoy his creation.

A third ‘dark’ view that was contested was that human rationality is too limited to count on and that we would do better to rely on traditional wisdom and divine revelation. Enlightened thinkers advocated the use of reason and pressed for investment in science and education.

This change of views had a great impact: it inspired revolutions and later practices for ‘planned social change’ in more peaceful and incremental ways. These changes were attended by a lively discourse on what is a good life and the social conditions required for it. The concept of happiness figured from the beginning in these discussions (Veenhoven 2015).

## Philosophy of Happiness

Happiness was a main topic in classical philosophy and interest in happiness revived in the eighteenth century Enlightenment (Mauzi 1960; Buijs 2007). The term ‘happiness’ was mostly used as an umbrella for various notions of the good life, which today is denoted as ‘quality of life’ and ‘well-being’. As such contemporary Social Indicators Research extends on the Enlightened philosophy of happiness. Since most philosophers earned their living as moral advisors, many tended to equate the good life with a morally good life. Today that orientation is most visible in Positive Psychology.

The term ‘happiness’, from the beginning, was also used in the more limited sense of subjective enjoyment of life, in other words as ‘life satisfaction’. Democritus (460-370 BC) was one of the Greek philosophers who addressed this meaning, while Jeremy Bentham (1748–1832) defined happiness as ‘the sum of pleasures and pains’.

Bentham (1789) articulated the difference between a morally good life and a pleasant life in his consequentionalistic ethic, which holds that good or bad should not be judged on fit with abstract moral principle, but by the reality of consequences on happiness, the morally best action being the one that produces the greatest happiness for the greatest number of people. In this view happiness should be the main aim of governments. A present day proponent of this view is Layard (2005).

Though welcomed in Enlightened circles in the eighteenth century, this view was rejected by the dominating ideologies of the nineteenth century and the first half of the twentieth century. The strongest opposition came from the churches, which preached a principalistic morality based on the biblical Ten Commandments. The liberals of that time also had reservations about the greatest happiness principle; in their power struggle with the aristocracy they preferred to emphasize freedom. The socialists who entered the scene in the late nineteenth century prioritized social equality. Nationalism dominated in first half of the twentieth century when the two world wars took place, and nationalists were more interested in national glory than in individual happiness.

As a result, interest in happiness declined and one of the indications is a sharp drop in the use of the word ‘happiness’ in book titles after 1800 (Buijs 2007). When I took a course in social philosophy in the 1960s, I found happiness described as a historical concern, not as a contemporary issue. But change was coming, in the bookshops I saw ever more of the ‘How to be happy’ type of self-help books and ‘happiness’ became a buzz-word in the media.

## Emergence of Empirical Happiness Research in the 1960s

This renewed interest in happiness in the second half of the twentieth century was driven by several factors. One of these is that many of the pressing ills had been overcome at that time, at least in the West. The era was characterized by peace, democracy and an unprecedented rise in the standard of living. This gave way to more positive goals, such as health and happiness. Another factor was the development of a multiple-choice-society in which individuals could choose how to live their life and therefore get interested in what way of life will be most satisfying. The rise of happiness on the political agenda was also facilitated by the weakening of the earlier ideological opposition mentioned above. The churches had declined in power, the liberals and the socialists achieved their main aims and nationalism had lost much of its appeal.

The effect of these long-term ideological shifts was amplified by technical developments and the development of empirical social science research, survey research in particular. Life-satisfaction is something we have in mind and as such it can be measured using self-reports. Hence happiness of a great number can be measured by including questions on life-satisfaction in large scale surveys among a general population. This has become common practice. Happiness is now a standard topic in many periodical social surveys, such as the American General Social Survey. This has yielded a lot of data on the basis of which initial qualms about the quality of responses to such questions have been tested. Though not free of measurement error, these questions appeared to do quite well (Diener 1994).

**Fig. 1:**
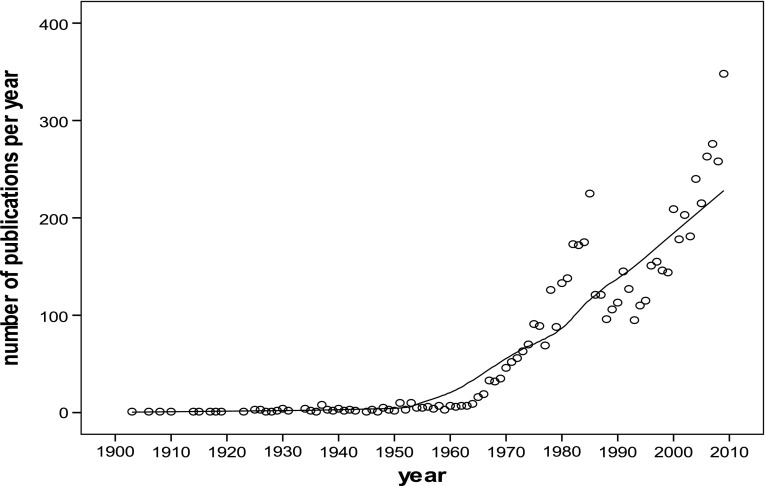
Number of scientific publications on happiness over time. *Source*: Bibliography of happiness (Veenhoven 2016)

The first surveys on happiness date from the late 1940s in the USA and were part of public opinion research (AIPO studies, cited in Easterlin 1974). In the 1950s happiness became a topic in research on successful aging (e.g. Kutner et al. 1956), in some studies on family life (e.g. Rose 1955) and in studies on work (e.g. Brayfield et al. 1957). In the 1960s happiness was used as an indicator in studies about mental health in the general population (e.g. Gurin et al. 1960). The number of scientific publications on happiness started to grow, as can be seen from Fig. 1. The numbers in Fig. 1 are not based on a count of publications that use the *word* happiness, but on publications that deal with the concept, however named. Fit with the concept of happiness as ‘subjective enjoyment of life’ was ascertained by close reading of the texts.

## Synergy of Happiness with Wider Social Indicators Research

The take-off of happiness research in the 1970s coincided with the emergence of the social indicators movement, which linked several existing strands of research. The political climate was favorable at that time, the post-war economic growth had resulted in the first wave of the ‘limits to growth’ movement, which today fall under the name ‘Beyond GDP’. Paradoxically, the contested economic affluence also provided the funds for research on alternative measures of social progress.

Institutes for Social Indicators Research appeared in most developed nations, sometimes government offices and sometimes based in universities. These institutes initiated large scale social surveys, for most of which happiness was, and continued to be, a topic. The results of such surveys are typically well covered by the media, findings on happiness in particular. While most strands in Social Indicators Research feed technocratic demand in the first place, data on happiness typically serves the general interest.

The simultaneous development of Social Indicators Research in various developed nations created a demand for international cooperation. This demand has been met in several ways. Firstly by the founding of an international journal ‘Social Indicators Research’ in 1974 by Alex Michalos. This journal paved the way for several more specialized journals (see Table 1) and by a book series.

**Table 1:** Publications on happiness in scientific journals on social indicators research and related movements. *Source*: Bibliography of happiness (Veenhoven 2016)

Year established	Name of journal	Discipline	Publications on happiness
1974	Social Indicators Research	Social sciences	702
1997	Quality of Life Research	Health sciences	131
2000	Journal of Happiness Studies	Interdisciplinary	450
2002	Health and Quality of Life Outcomes	Health sciences	5
2003	Journal of Positive Psychology	Psychology	27
2003	Applied Quality of Life Research	Social sciences	39
2011	Psychology of Wellbeing	Psychology	4
2011	International Journal of Wellbeing	Interdisciplinary	8
2012	International Journal of Happiness and Development	Interdisciplinary	15

Happiness research has profited much from these new outlets, and my personal experience can serve as an illustration. When I entered the field in the 1980s several of my papers on happiness were rejected by sociological journals, typically because reviewers considered the subject unscientific. Fortunately, Social Indicators Research did accept papers on the matter. Likewise, my book ‘Conditions of Happiness’ (Veenhoven 1984, still available), was initially rejected by Reidel Publishing (now Springer) on the advice of the philosopher in charge, but was accepted when passed on to the advisor for Social Indicators Research for comment.

Happiness became an ever more prominent topic in the journal Social Indicators Research and for that reason a more focused journal was established in 2000, the Journal of Happiness Studies. Alex Michalos, founding father of Social Indicators Research, was one of the founding editors.

Happiness research has also profited from the organizational infra-structure that developed around the Social Indicator Movement. In 1988 Alex Michalos initiated the first international research association, the Working Group Social Indicators (WG04) of the International Sociological Association (ISA), which was upgraded to a research committee (RG55) in 2008. In 1995 an interdisciplinary research association was founded, the International Society for Quality of Life Studies (ISQOLS). The rapid institutionalization of Social Indicators Research is described in more detail by Land & Michalos (2017). Happiness research now had a podium and organized channels of contacts and information. The brood chamber of Social Indicators Research also helped happiness research to keep its focus on social policy and curbed the tendency to become lost in spirituality.

## Place of Happiness in Related Social Scientific Movements

Happiness is a topic in three other tracks in the social sciences that developed parallel to Social Indicators Research.

The first of these is research on ‘Health Related Quality of Life’ (HQOL), that is, research on patient reported outcomes of disease and medical treatment. This research line draws on inspiration similar to that of Social Indicators Research, in this case, ‘beyond mere survival’ instead of ‘beyond GDP’. ‘Quality’ of life is mostly measured using indicators of health, but sometimes with happiness. Developments in the technique of experience sampling will probably make this topic more prominent in the future.

Another scientific track parallel to Social Indicators Research is ‘Positive Psychology’. Positive Psychology is about positive mental health and goes ‘beyond mental disorder’. The movement serves the counseling and education professions. Happiness is a topic in this sphere, but not the most prominent one. The focus is not on life-satisfaction (called ‘hedonic’ happiness in these circles), but on a fuzzy set of personality characteristics deemed desirable, such as autonomy and self-acceptance. This syndrome is called ‘eudaimonic’ happiness, but in my view ‘positive mental health’ is a better word. The traits involved fit the first description of positive mental health by Jahoda (1959). Happiness (hedonic) is on her list.

The most recent development in happiness research is the emergence of ‘Happiness Economics’, which can be seen as a social indicator movement within economics. A key insight in Happiness Economics is that the satisfaction we expect from a choice (expected utility) does not always fit the satisfaction that actually results from the choice (experienced utility) and that we can choose more rationally if better informed about the probable effects on satisfaction. In Happiness Economics the focus is typically on happiness in the sense of life-satisfaction.

The place of happiness in Social Indicators Research and in parallel scientific movements is illustrated by the number of publications on this subject in related scientific journals. See the overview given in Table 1. As for Fig. 1, the number of publications on happiness is not assessed on the basis of use of the *word* ‘happiness’, but on close reading of the texts to assess whether the *concept* is addressed, that is, subjective enjoyment of one’s life as a whole. The development of the field is also nicely illustrated by the years in which these journals were established. An overview of the research associations in which happiness is a topic is provided in Table 2 and illustrates the same development over time.

**Table 2:** Research associations in which happiness is a topic

Year established	Name	Discipline
1988	Research Committee on Social Indicators Research (RC55) of the International Sociological Association (ISA)	Sociology
1993	International Association for Quality of Life Research (ISOQOL)	Health sciences
1995	International Society for Quality of Life Studies (ISQOLS)	Social sciences
2002	European Network for Positive Psychology (ENPP)	Psychology
2007	International Association for Positive Psychology (IPPA)	Psychology
2016	Network for Happiness Economics	Economics
